# Artificial Neural Network Modeling of Novel Coronavirus (COVID-19) Incidence Rates across the Continental United States

**DOI:** 10.3390/ijerph17124204

**Published:** 2020-06-12

**Authors:** Abolfazl Mollalo, Kiara M. Rivera, Behzad Vahedi

**Affiliations:** 1 Department of Public Health and Prevention Sciences, School of Health Sciences, Baldwin Wallace University, Berea, OH 44017, USA; krivera19@bw.edu; 2 Department of Geography, University of California Santa Barbara (UCSB), Santa Barbara, CA 93106, USA; behzad@ucsb.edu

**Keywords:** artificial neural networks, COVID-19 (Coronavirus), GIS, multilayer perceptron, United States

## Abstract

Prediction of the COVID-19 incidence rate is a matter of global importance, particularly in the United States. As of 4 June 2020, more than 1.8 million confirmed cases and over 108 thousand deaths have been reported in this country. Few studies have examined nationwide modeling of COVID-19 incidence in the United States particularly using machine-learning algorithms. Thus, we collected and prepared a database of 57 candidate explanatory variables to examine the performance of multilayer perceptron (MLP) neural network in predicting the cumulative COVID-19 incidence rates across the continental United States. Our results indicated that a single-hidden-layer MLP could explain almost 65% of the correlation with ground truth for the holdout samples. Sensitivity analysis conducted on this model showed that the age-adjusted mortality rates of ischemic heart disease, pancreatic cancer, and leukemia, together with two socioeconomic and environmental factors (median household income and total precipitation), are among the most substantial factors for predicting COVID-19 incidence rates. Moreover, results of the logistic regression model indicated that these variables could explain the presence/absence of the hotspots of disease incidence that were identified by Getis-Ord Gi* (*p* < 0.05) in a geographic information system environment. The findings may provide useful insights for public health decision makers regarding the influence of potential risk factors associated with the COVID-19 incidence at the county level.

## 1. Introduction

Novel coronavirus disease (COVID-19) has rapidly spread worldwide, becoming a global health threat [1]. The disease was first identified in Wuhan, China, and continued to spread out across the world [2]. According to the World Health Organization [3], as of 4 June 2020, there have been more than 6.4 million confirmed cases and over 380 thousand deaths worldwide. These statistics have surpassed the number of deaths and cases for Middle East respiratory syndrome (MERS) and severe acute respiratory disorder (SARS) since their outbreaks [4]. The pandemic has directly impacted the economy, society, and healthcare systems. According to the International Monetary Fund [5], global economic growth in the year 2020 is estimated to be -3.0%, compared to +2.9% in 2019. The United Nations predicts that the pandemic can continue to adversely impact societies with perpetual disease spread due to improper policy interventions [6].

Although the United States is ranked number one in the global health security index [7], it is the leading country in the number of confirmed cases and deaths globally [8]. As of 4 June 2020, there have been over 1.8 million confirmed cases and more than 108,000 deaths in this country [9]. Moreover, the case fatality ratio (CFR) continues to fluctuate in this country. As of 4 June 2020, the United States ranks in ninth place worldwide, with a CFR of 5.8% [10].

Recent studies have demonstrated that preexisting conditions, such as cardiovascular diseases [11], respiratory diseases [12], cancer [13], infectious diseases [14], and substance abuse [15], can contribute to the elevated morbidity and mortality of COVID-19. In China, Zheng et al. [11] utilized the MERS virus as a reference and suggested that SARS-CoV-12 can cause cardiac failure and acute myocarditis. Although the findings were preliminary, they indicated that patients could experience chronic cardiovascular effects secondary to contracting the disease. Lippi and Henry [12] conducted a meta-analysis demonstrating that chronic obstructive pulmonary disease (COPD) patients are five times more at risk of contracting the SARS-CoV-2 virus. You et al. [13] alluded to the guidelines suggested by French medical oncologists on cancer patient care during the pandemic. In South Africa, Cox et al. [14] highlighted changes in tuberculosis (TB) patients’ treatment during the pandemic. In the United Kingdom, Marsden et al. [15] indicated how individuals with substance abuse disorders might experience addiction augmentation during the pandemic, consequently, increasing the risk for COVID-19 contraction. They suggested that substance abuse disorder may not be overlooked when addressing preexisting conditions in COVID-19 patients.

In addition to preexisting conditions, environmental [16], demographic, and socioeconomic [17] factors can potentially influence COVID-19 incidence. For instance, Wang et al. [16] indicated that COVID-19 transmission is influenced by temperature variability. Their results suggest that reduced COVID-19 transmission is associated with higher humidity and temperature. In the United States, Mollalo et al. [17] suggested that higher percentages of nurse practitioners and black females and higher income inequality at the county level could explain 68.1% of COVID-19 incidence geographic variations. 

Artificial neural networks (ANNs) are relatively novel techniques to model complex non-linear relationships in spatial epidemiology [18]. The techniques have been applied in a variety of fields, including but not limited to environmental science [19,20], agriculture [21], finance [22,23], artificial intelligence [24], epidemiology and public health [25,26,27]. Reddy and Imler [26] demonstrated that ANNs could provide reliable predictions for chronic diseases, such as cirrhosis patients with hepatocellular carcinoma. They found high sensitivity (80.61–86.67%) and specificity (99.88–99.95%), corresponding to demographic and physiological inputs. Badnjević et al. [28] incorporated ANNs to classify asthma; they found high levels of sensitivity (97.11%) in asthmatic individuals and specificity (98.85%) in healthy individuals. Their findings suggested that ANNs can be appropriate techniques for asthma detection. Due to a lack of research on the spatial complexities of COVID-19 at the national level, in this study, we leveraged the potential of ANNs in identifying complex spatial patterns and the power of geographic information systems (GIS) in spatial analysis [29,30] to predict county-level COVID-19 incidence rates in the continental United States. We employed one of the widely used topologies of ANNs that is described in Section 2.4.

## 2. Materials and Methods

### 2.1. Data Collection and Preparation

COVID-19 is continually monitored by governmental health agencies and institutions of higher learning, such as the US Centers for Disease and Control and Johns Hopkins University [31]. In this study, we compiled a database of 57 candidate variables that may predict county-level cumulative disease incidence as a dependent variable. From January 22 to April 25, 2020, cumulative numbers of confirmed cases of COVID-19 across the continental United States were collected at the county level from USAFacts (usafacts.org) and normalized by populations. The counties (*n* = 3109) were considered as samples that represent the status of the disease in the US. In this study, socioeconomic (such as household income, income inequalities, and unemployment rate), behavioral (such as smoking), environmental (such as temperature, precipitation, and air pollution), topographic (such as altitude, and terrain slope), and demographic (such as proportions of age groups, race, gender, and access to primary care) factors were prepared at the county level and were used as explanatory variables. To avoid reiteration, a complete description of the used variables has been provided in Mollalo et al. [17].

In addition to the above explanatory variables, which were also used in the study of Mollalo et al. [17], age-adjusted mortality rates of several diseases were incorporated, including infectious diseases (i.e., TB, HIV/AIDS, hepatitis, and lower respiratory infection), cardiovascular diseases (i.e., cerebrovascular disease, hypertensive heart disease, ischemic heart disease, cardiomyopathy and myocarditis, atrial fibrillation and peripheral vascular disease), chronic respiratory diseases (i.e., COPD, asthma, interstitial lung disease, and pulmonary sarcoidosis), cancer (i.e., pancreatic, gallbladder and biliary tract, mesothelioma, Hodgkin lymphoma, leukemia, tracheal, bronchus, and lung cancer), and substance use disorders (i.e., drug and alcohol use). The data were retrieved from the University of Washington Global Health Data Exchange (http://ghdx.healthdata.org/us-data) and joined to the preexisting database. All data were collected and prepared at the county level and are publicly available. A list of all variables can be found in the Supplementary Materials.

### 2.2. Spatial Analysis

We examined the geographic distribution of the COVID-19 incidence rate using global and local indices. The global Moran’s index [32,33] was used to identify the overall pattern (random, clustered, or dispersed) of disease incidence rate using the following formula:

(1) $$I=\frac{n\;\sum_{i=1}^{n}\sum_{j=1,\;j\neq i}^{n}w_{ij}C_{i}C_{j}}{\sum_{i=1}^{n}\sum_{j=1}^{n}w_{ij}\sum_{i=1}^{n}C_{i}^{2}}$$

where $C_{i}$ and $C_{j}$ are the deviations of COVID-19 incidence rates from the mean incidence rate for county i and county j, respectively; $w_{ij}$ is the spatial weight between county i and county j, which is non-zero when the counties are neighbors (i.e., share borders); and n is the total number of counties. The value of I ranges between −1 and +1. The values close to 0 indicate random distribution (null hypothesis), while values close to +1 and −1, respectively, indicate positive and negative spatial autocorrelations [34,35].

As the global Moran’s index is unable to identify the location of hotspots [35], Getis–Ord G_i_*, statistics developed by Getis and Ord [36] were used to identify the hotspots of COVID-19 incidence rates (*p* < 0.05) as follows [37]:

(2) $$G_{i}^{*}=\frac{\sum_{j=1}^{n}w_{ij}C_{j}-\bar{C}\sum_{j=1}^{n}w_{ij}}{S\;\sqrt{\frac{\left[n\sum_{j=1}^{n}w_{ij}^{2}-{\left(\sum_{j=1}^{n}w_{ij}\right)}^{2}\right]}{n-1}}}$$

(3) $$S=\sqrt{\frac{\sum_{j=1,j\neq i}^{n}{C_{j}}^{2}}{n-1}-{\bar{C}}^{2}}$$

The positive and high value of $G_{i}^{*}$ indicates a more intense clustering of high values (hotspot(s)). The output of the $G_{i}^{*}$ statistic was mapped in ArcGIS 10.7 (Esri, Redlands, CA, USA) to locate the hotspots of COVID-19 incidence rates.

### 2.3. Feature Selection

The presence of a relatively large number (*n* = 57) of potentially relevant variables can create a technical problem and a theoretical discrepancy, which can in turn decrease the generalizability of the neural networks [38]. Therefore, we applied the Boruta algorithm [39] to identify feature importance, and ultimately chose “all-relevant” important features [40]. This algorithm is a wrapper around the Random Forest classification algorithm and is implemented in the “Boruta” package in *R*. To determine important and unimportant features, this algorithm creates random shadow variables and runs a random forest classifier on the set of original and shadow variables. Based on the results of a statistical test (using z-scores), the algorithm iteratively removes the variables that have lower z-scores compared to the shadow variables [39]. After performing the Boruta feature selection algorithm and also Pearson’s correlation analysis on the training dataset, important and less correlated (r < 0.7) variables were identified and selected as input variables in the neural networks.

### 2.4. Artificial Neural Networks

Artificial neural networks (ANNs) are computational structures that can learn the relationship between a set of input and output variables through an iterative learning process. These networks use simple computational operations such as addition and multiplication, yet they are capable of solving complex, non-linear problems [41,42,43]. Once a network is properly trained, it can be used to predict a variable of interest based on an independent (holdout) dataset, usually with minimal modifications [44].

The main components of ANNs are neurons that are organized in layers and are fully connected to the next layer by a set of weights (edges). Each ANN consists of one input layer, one output layer, and at least one hidden layer. The simplest form of ANN is called a perceptron, first introduced by Rosenblatt [45], which is the building block of neural networks. In a perceptron, each input is multiplied by a corresponding weight and then aggregated by a mathematical function called “activation of the neuron.” Another function then computes the output. ANNs are a set of layers that are created by stacking perceptrons. For instance, if the inputs to the *i*th perceptron in a network are denoted by $x_{1i},\ldots ,\;x_{ni}$, assuming that a summation function is used to calculate the outputs (denoted by $z_{i}$), we will have [44]:

(4) $$z_{i}=\overset{m}{\underset{j=1}{\sum }}x_{ij}w_{ij}+b_{i}$$

where *n* is the number of inputs; *m* is the number of neurons in the current layer; $w_{ij}$ is the weight of the jth neuron (jth input to the ith cell), and $b_{i}$ is a bias term. In matrix form, $z_{i}$ can be simplified to:

(5) $$z_{i}=w_{i}^{T}x_{i}+b_{i}$$

where

(6) $$w_{i}={\left[w_{i1},w_{i2},\;\ldots ,\;w_{in}\right]}^{T}$$

(7) $$b_{i}={\left[b_{i1},b_{i2},\;\ldots ,\;b_{in}\right]}^{T}$$

Given a specific loss function, the perceptron can reach better estimates of the output values by adjusting the weights and bias terms through an iterative process referred to as error-correction learning. This process calculates the “errors” using observed and estimated values and “corrects” network parameters based on those errors. Given the estimated value of the network output at iteration *n*, (i.e., $d_{n}$), and the observed output value $y_{n}$, a loss term is defined by [46]:

(8) $$L\left(n\right)=Loss\left(d_{n},\;y_{n}\right)$$

where *Loss* is a function of $d_{n}$ and $y_{n}$, which gives a measure of the difference between observed and estimated output values and is defined based on the type of problem. This *Loss* term can be used locally at each neuron to update the weights of the network (in that neuron) using gradient descent learning:

(9) $$w_{ij}\left(n+1\right)=w_{ij}\left(n\right)-\eta \;\frac{\partial \;L\left(n\right)}{\partial w_{ij}\left(n\right)}$$

where, at iteration *n*, $w_{ij}$ is the weight from neuron j to neuron i, η is the step size, and $\frac{\partial \;L\left(n\right)}{\partial w_{ij}\left(n\right)}$ is the partial derivative (gradient) of *Loss* with respect to $w_{ij}$. Step size is one of the (hyper) parameters of a network and can be optimized by trial and error. A similar procedure is used to update bias terms.

The activation function is a non-linear function applied to each neuron to transfer its values into a known range, for instance, [−1, 1] or [0, 1]. The most common activation functions in ANNs are rectified linear unit (ReLU), sigmoid, and hyperbolic tangent (tanh) [47]. The summation term in Equation 4 acts as an activation function for the perceptron.

(10) $$\sigma \left(z\right)=\frac{1}{1+e^{-z}}\;\;\left(sigmoid\right)$$

(11) $$tanh\left(z\right)=\frac{2}{1+e^{-2z}}-1$$

(12) $$ReLU\left(z\right)=\{\begin{array}{c} z\;\;\;\;if\;z>0\\ 0\;\;\;\;if\;z\leq 0 \end{array}$$

**Figure 1:**
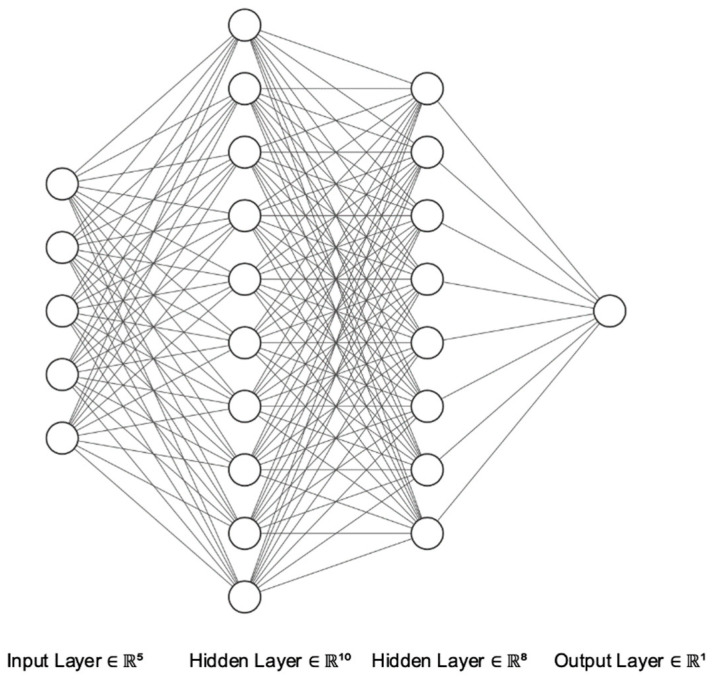
The topology of MLP neural network.

In this study, the performance of multilayer perceptron (MLP) neural networks in modeling the disease incidence is investigated across the continental United States. MLP is a variant of the (single) perceptron model explained above and is one of the most popular classes of feedforward ANNs, with one or more hidden layers between the input and output layers [48]. MLP is used in supervised learning tasks for classification or regression. Figure 1 represents the topology of the MLP neural network. In this regression study, we employed MLP with 1 and 2 hidden layers. The “Neuralnet” package in R was used to train the MLP.

### 2.5. Model Performance 

The entire dataset was randomly divided into three different categories: 1) training samples: 60% (*n_t_* = 1865) of data used for developing the models; 2) cross-validation samples: 15% (*n_c_* = 466) of data used to fine-tune network weights and to avoid overfitting; 3) holdout samples: 25% (*n_h_* = 777) of data used to test the accuracy and generalizability of the models. The same partitioned data were used for all models for the purpose of comparison. The process of training models stopped at earlier stages to avoid overfitting. The performances of neural networks in predicting COVID-19 cumulative incidence rate (output) based on selected variables (inputs) were compared to each other, and to the linear regression model as a baseline on holdout samples. We used three different evaluation measures for accuracy assessments: root-mean-square error (RMSE), mean absolute error (MAE), and the correlation coefficient between observed COVID-19 incidence rate and model predictions (r). In this study, the model with minimum error values and a higher correlation coefficient was considered as the optimal model [47]. Below are the formulae to assess the accuracies:

(13) $$RMSE=\sqrt{\frac{\sum_{i=1}^{n}{\left(O_{i}-P_{i}\right)}^{2}}{n}}$$

(14) $$MAE=\frac{1}{n}\overset{n}{\underset{i=1}{\sum }}|O_{i}-P_{i}|$$

(15) $$r=\frac{\sum_{i=1}^{n}(O_{i}-\bar{O})(P_{i}-\bar{P})}{\sqrt{\sum_{i=1}^{n}{\left(O_{i}-\bar{O}\right)}^{2}\sum_{i=1}^{n}{\left(P_{i}-\bar{P}\right)}^{2}}}$$

where $O_{i}$ is the observed value of the COVID-19 incidence rate, $P_{i}$ is the predicted value by the model, and n is the number of observations on a holdout dataset.

Sensitivity analysis was carried out on the optimal model to assess the contributions of variables in predicting disease incidence. Finally, vanilla logistic regression was utilized to explain the relationship of the most contributing factors obtained from sensitivity analysis and the presence/absence of hotspots identified by Getis-Ord G_i_*.

## 3. Results

**Figure 2:**
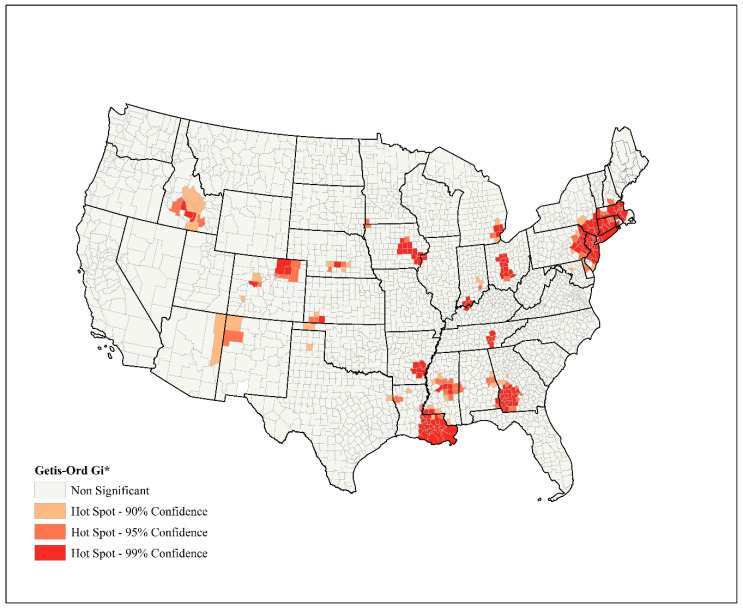
Locations of hotspots of COVID-19 incidence identified by Getis-Ord G_i_*, continental United States.

Results of spatial analysis with Global Moran’s *I* indicated that the distribution of COVID-19 incidence rate in the continental United States is clustered (Index: 0.36, z-score: 34.75, *p* < 0.0001), rejecting the null hypothesis (random distribution). Moreover, Getis-Ord G_i_* could identify the location of hotspots of disease incidence rates (Figure 2). In total, 217 counties were identified as hotspots (*p* < 0.05), which were mainly located in the northeastern regions of the continental United States, western Georgia, central Ohio, southern Louisiana, and northeast Iowa.

**Figure 3:**
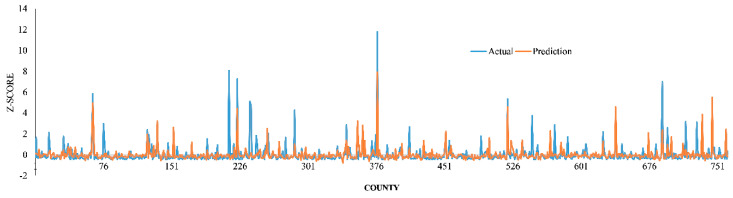
Comparison of actual and predicted values of the dependent variable (z-scores) for holdout samples using the one-hidden-layer MLP.

The Boruta algorithm and Pearson’s correlation analysis selected 34 variables as less correlated and important variables (Supplementary Materials), which were then fed as inputs to ANNs. Overall, among the activation functions, “tanh” had slightly better performance (lowest RMSE) and thus was used in the MLPs. We systematically increased the number of neurons in the hidden layers from 10 to 30. The lowest errors were obtained with 15 neurons in the hidden layer. The performances of all employed models, in terms of RMSE, MAE, and r between observed COVID-19 incidence rate and model predictions on the holdout sample are presented in Table 1. Correlation coefficients of the models ranged between 0.30 and 0.65. The linear regression model achieved the least correlations with observed COVID-19 incidence rates (r < 0.3). On the contrary, the MLP with one hidden layer achieved the highest correlation (r = 0.65), indicating a satisfactory agreement between model predictions and observed COVID-19 incidence rates. Moreover, the accuracy assessment of the results indicated that the prediction error of the MLP with one hidden layer is less than others (RMSE = 0.72, MAE = 0.36). The worst performance was obtained by linear regression (RMSE = 0.99, MAE = 0.58), while the MLP with one hidden layer yielded better accuracy and generalization capability than other models and was thus considered as the proposed model for further analysis. Figure 3 compares the z-scores of actual and predicted values of the dependent variable for holdout samples using the one-hidden-layer MLP.

**Figure 4:**
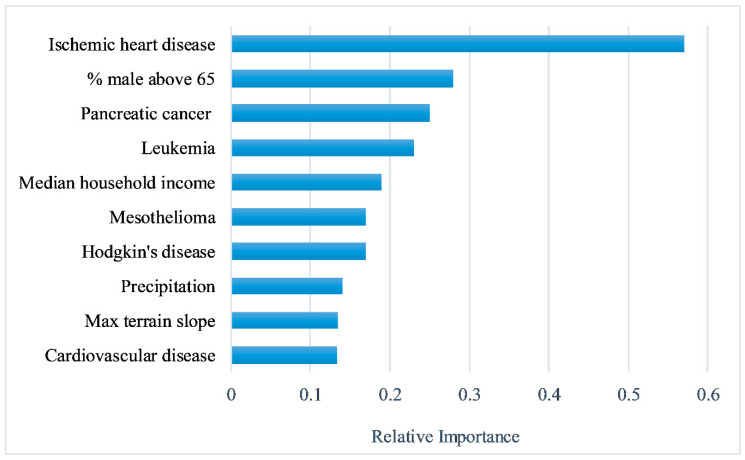
The relative importance of the top 10 variables to the COVID-19 incidence rate, using sensitivity analysis by one hidden layer MLP, continental United States.

We performed a sensitivity analysis to investigate the effect of each variable on the COVID-19 incidence rate using the MLP with one hidden layer. Figure 4 shows the top 10 contributing variables in order of importance. According to Figure 4, age-adjusted mortality rates of ischemic heart disease, pancreatic cancer, leukemia, Hodgkin’s disease, mesothelioma, and cardiovascular disease were among the top 10 factors with the highest relative importance for COVID-19 incidence rates, showing the potential importance of these preexisting conditions to COVID-19 incidence rate. In addition to the mortality rates, the proportion of males above 65 years old, higher median household income, precipitation, and maximum terrain slope were other important contributing variables.

The logistic regression model was used to explain the association between the presence/absence of the identified hotspots (*p* < 0.05) of COVID-19 incidence rates and the explanatory variables obtained from sensitivity analysis. The results indicate that age-adjusted pancreatic cancer mortality rates followed by median household income, precipitation, and Hodgkin’s disease mortality rates could explain the positive association with the presence/absence of hotspots. Meanwhile, age-adjusted mortality rates for leukemia and cardiovascular disease, and maximum terrain slope, were negatively correlated with the occurrence of the hotspots. Table 2 summarizes the results of the logistic regression model statistics.

## 4. Discussion

COVID-19 is an RNA virus that has the potential to mutate like the flu and measles, which may have contributed to the rapid transmission of the disease [49]. Due to the successful performance of ANNs in modeling many complex relationships, we examined the applicability of ANNs in predicting COVID-19 incidence in the continental United States. One of the main advantages of ANNs over widely applied traditional statistical techniques is their predictive capabilities even when working with noisy, complex, and incomplete datasets [18], which may also be useful for modeling other viruses with complex epidemiology, such as Zika virus. This motivated us to compile a relatively broad range (*n* = 57) of socioeconomic, behavioral, environmental, topographic, and demographic factors together with mortality rates of preexisting conditions. The variables were either suggested by previous studies or were based on domain knowledge (rarely investigated at the county level).

Among the different combinations of network topologies and learning parameters that were examined, the MLP with one hidden layer performed better and thus was used for predictions. Sensitivity analysis of this model indicated that six age-adjusted mortality rates, including ischemic heart disease, pancreatic cancer, leukemia, Hodgkin’s disease, mesothelioma, and cardiovascular disease, had substantial impacts on county-level COVID-19 incidence across the continental United States. While there is still much to discover and research, the results suggest that the disease incidence may be influenced by the fluctuance in mortality rates’ distribution nationwide. Therefore, counties with elevated proportions of mortality rates of one or more chronic conditions may be more vulnerable to the higher incidence of COVID-19, when compared to other counties. As a result, it may potentially impact mortality rates during the pandemic. Lai et al. [50] indicated that comorbidities and cancer might be substantial contributors to COVID-19 mortality excess rates. They proposed that their findings are applicable to COVID-19 incidence and mortality in the United States. Hanff et al. [51] convey that COVID-19 mortality is significantly associated with comorbidities, including cardiovascular diseases (i.e., hypertension), suggesting that further studies may focus on detailed descriptions of comorbid physiological implications in COVID-19 patients, especially in the use pharmacological therapies. Alimadadi et al. [52] proposed that sophisticated analysis, such as machine learning and artificial intelligence, may aid in combating the pandemic. They also suggest that these methods may provide a better understanding of COVID-19 diagnosis, medication treatment, prevention, and hospital logistics. Although our findings seem consistent with recent studies, drawing conclusions at the individual level is not valid due to ecological fallacy, thus the findings can only be interpreted at the county level.

According to our findings, demographic (i.e., % male above 65), socioeconomic (i.e., median household income), and environmental factors (i.e., maximum terrain slope and precipitation) are influential in predicting COVID-19 incidence, indicating that the disease is not merely affected or driven by physiological conditions. The findings support and extend the previous study of Mollalo et al. [17], who utilized multiscale geographically weighted regression to explain geographic county-level variations of COVID-19 incidence in the United States. Their results indicated that counties with higher median household income and income inequalities were positively correlated with elevated disease incidence, predominantly in the tristate area. Kavanagh et al. [53] proposed that socioeconomic and demographic factors are vital to consider when addressing the pandemic as they may be associated with income disparities that exist in the United States. This may be the case of some employees that may not have the option to work remotely from home, instead, potentially resulting in more frequent exposure to the virus, contributing to further spread of the disease. The study of Qu et al. [54] emphasize the significance of examining the effects of environmental factors pertaining to COVID-19. Their results suggest that COVID-19 may be aggravated by air pollutants (i.e., airborne particulate matter), influencing infectivity. Hence, further studies on preexisting conditions, socioeconomic, demographic, and environmental impacts on COVID-19 incidence preferably at a less coarse granularity level are essential.

We acknowledge that the obtained consistency between the model and ground truth is not notably large. This is likely due to the limited knowledge about the recently emerged disease and factors that may be influential but not included in this study. Therefore, future studies should focus on improving the prediction accuracy of this initial model. Additionally, even though no significant difference is observed between the performance of MLP networks with one and two hidden layers, there may still exist complex relationships in the data that are not captured. This leads us to another limitation of this study, which is the number of training samples. With a higher amount of training data, one could apply deeper networks, i.e., networks with more than two hidden layers, and leverage the power of deep learning models. Deeper neural networks can capture potential non-linearity in the relationship between dependent and independent variables by stacking two or more hidden layers. Thus, such networks are, in general, capable of reaching higher accuracies and can reveal the nuances of the data. However, the amount of training data that was available in this study does not justify utilizing deep networks. A few possible solutions to increase the amount of data are to consider a longer temporal interval (which was not possible in this case), to incorporate data from other countries and regions, to use finer spatial units data (if available), or to use data augmentation techniques to (artificially) generate more training data and features. Moreover, although adjusted mortality rates of the diseases used in this study cannot be directly interpreted as preexisting conditions, higher mortality rates of a certain disease could allude to a higher incidence rate of it. Therefore, this study could be used to further investigate any potential correlation between disease prevalence and COVID-19 incidence.

After more than three months since the first confirmed case of COVID-19 in the US, and due to the substantial economic and social impacts of the pandemic itself and the resulting lockdown policies, discussions regarding “re-opening the country” are omnipresent. The findings of this paper could be used as one of the many guidelines needed by policymakers to decide if and where (at the county level) lockdown policies should be relaxed.

## 5. Conclusions

In this study, we examined the applicability of multi-layer perceptron artificial neural networks in modeling cumulative incidence of COVID-19 at the county-level across the continental United States. Although the employed model indicated a reasonable but not large consistency with ground-truth on holdout samples, the prediction capability of the model requires a significant improvement possibly by incorporating new related variables or perhaps by employing different machine learning algorithms. However, with the obtained accuracy, (age-adjusted) mortality rates of ischemic heart disease, pancreatic cancer, leukemia, Hodgkin’s disease, mesothelioma, and cardiovascular disease together with two socioeconomic and environmental factors (median household income and total precipitation) could contribute with the disease incidence. Therefore, further studies of the factors and their associations with the disease may reveal useful information for monitoring COVID-19 outbreak.

## Figures and Tables

**Table 1:** Comparative performance of the employed models (single run) to predict COVID-19 rates across the continental United States.

Model	Accuracy Assessment		
	RMSE	r	MAE
Linear Regression	0.992517	0.295885	0.577808
MLP (1 hidden layer)	0.722409	0.645481	0.355843
MLP (2 hidden layers)	0.839806	0.466981	0.39755

**Table 2:** Results of the logistic regression model in explaining the presence/absence of the hotspots (*p* < 0.05) of COVID-19 incidence rate, continental United States.

	Coefficient (B)	Standard Error	Wald Test	Degree of Freedom	Significance	Exp (B)
Constant	−2.763	0.086	1036.109	1	0.000	0.063
Median household income	0.403	0.079	26.139	1	0.000	1.497
Max terrain slope	−0.270	0.093	8.432	1	0.004	0.763
Precipitation	0.337	0.080	17.817	1	0.000	1.400
Pancreatitis cancer	0.636	0.095	44.672	1	0.000	1.889
Hodgkin’s Disease	0.409	0.100	16.596	1	0.000	1.505
Leukemia	−0.550	0.089	38.241	1	0.000	0.577
Cardiovascular	−0.414	0.118	12.350	1	0.000	0.661

## Supplementary Materials

The following are available online at https://www.mdpi.com/1660-4601/17/12/4204/s1.

## References

[1] Fauci A.S., Lane H.C., Redfield R.R. (2020). Covid-19—Navigating the Uncharted. N. Engl. J. Med..

[2] World Health Organization WHO Timeline—COVID-19. https://www.who.int/news-room/detail/27-04-2020-who-timeline---covid-19.

[3] World Health Organization WHO Coronavirus Disease (COVID-19) Dashboard. https://covid19.who.int.

[4] National Institutes of Health COVID-19, MERS & SARS. https://www.niaid.nih.gov/diseases-conditions/covid-19.

[5] International Monetary Fund (IMF) World Economic Outlook Chapter 1: The Great Lockdown. https://www.imf.org/en/Publications.

[6] United Nations Everyone Included: Social Impact of COVID-19. https://www.un.org/development/desa/dspd/everyone-included-covid-19.html.

[7] Cameron E.E., Nuzzo J.B., Bell J.A. (2019). Global Health Security Index: Building Collective Action and Accountability.

[8] Johns Hopkins University Center for System Science and Engineering COVID-19 Dashboard. https://coronavirus.jhu.edu/map.html.

[9] The COVID Tracking Project. https://covidtracking.com/data/us-daily.

[10] Johns Hopkins University & Medicine Mortality Analyses. https://coronavirus.jhu.edu/data/mortality.

[11] Zheng Y.Y., Ma Y.T., Zhang J.Y., Xie X. (2020). COVID-19 and the cardiovascular system. Nat. Rev. Cardiol..

[12] Lippi G., Henry B.M. (2020). Chronic obstructive pulmonary disease is associated with severe coronavirus disease 2019 (COVID-19). Respir. Med..

[13] You B., Ravaud A., Canivet A., Ganem G., Giraud P., Guimbaud R., Kaluzinski L., Krakowski I., Mayeur D., Grellety T. (2020). The official French guidelines to protect patients with cancer against SARS-CoV-2 infection. Lancet Oncol..

[14] Cox V., Wilkinson L., Grimsrud A., Hughes J., Reuter A., Conradie F., Nel J., Boyles T. (2020). Critical changes to services for TB patients during the COVID-19 pandemic. Int. J. Tuberc. Lung Dis..

[15] Marsden J., Darke S., Hall W., Hickman M., Holmes J., Humphreys K., Neale J., Tucker J., West R. (2020). Mitigating and learning from the impact of COVID-19 infection on addictive disorders. Addiction.

[16] Wang J., Tang K., Feng K., Lv W. (2020). High temperature and high humidity reduce the transmission of COVID-19. Available SSRN.

[17] Mollalo A., Vahedi B., Rivera K.M. (2020). GIS-based spatial modeling of COVID-19 incidence rate in the continental United States. Sci. Total Environ..

[18] Mollalo A., Mao L., Rashidi P., Glass G.E. (2019). A GIS-based artificial neural network model for spatial distribution of tuberculosis across the continental United States. Int. J. Environ. Res. Public Health.

[19] Keshavarzi A., Sarmadian F., Sadeghnejad M., Pezeshki P. (2010). Developing pedotransfer functions for estimating some soil properties using artificial neural network and multivariate regression approaches. ProEnviron. Promediu.

[20] Marohasy J., Abbot J. (2015). Assessing the quality of eight different maximum temperature time series as inputs when using artificial neural networks to forecast monthly rainfall at Cape Otway, Australia. Atmos. Res..

[21] Abdipour M., Younessi-Hmazekhanlu M., Ramazani S.H.R. (2019). Artificial neural networks and multiple linear regression as potential methods for modeling seed yield of safflower (*Carthamus tinctorius L.*). Ind. Crop. Prod..

[22] Bae J.K. (2012). Predicting financial distress of the South Korean manufacturing industries. Expert Syst. Appl..

[23] Gordon R. (2019). Applications of Artificial Neural Networks in Financial Market Forecasting. Ph.D. Thesis.

[24] Kang B.H., Bai Q. (2016). AI 2016: Advances in Artificial Intelligence. Proceedings of the 29th Australasian Joint Conference.

[25] Kiang R., Adimi F., Soika V., Nigro J., Singhasivanon P., Sirichaisinthop J., Leemingsawat S., Apiwathnasorn C., Looareesuwan S. (2006). Meteorological, environmental remote sensing and neural network analysis of the epidemiology of malaria transmission in Thailand. Geospat. Health.

[26] Reddy R., Imler T.D. (2017). Artificial Neural Networks are Highly Predictive for Hepatocellular Carcinoma in Patients with Cirrhosis. Gastroenterology.

[27] Mollalo A., Sadeghian A., Israel G.D., Rashidi P., Sofizadeh A., Glass G.E. (2018). Machine learning approaches in GIS-based ecological modeling of the sand fly *Phlebotomus papatasi*, a vector of zoonotic cutaneous leishmaniasis in Golestan province, Iran. Acta Trop..

[28] Badnjević A., Gurbeta L., Cifrek M., Marjanovic D. (2016). Classification of asthma using artificial neural network. MIPRO, Proceedings of the International Convention, Proceedings of the 2016 39th International Convention on Information and Communication Technology, Electronics and Microelectronics (MIPRO), Opatija, Croatia, 30 May–3 June 2016.

[29] Allen C., Hervey T., Lafia S., Phillips D.W., Vahedi B., Kuhn W. (2016). Exploring the notion of spatial lenses. Geographic Information Science, Proceedings of the Annual International Conference on Geographic Information Science, Cham, Switzerland, September 2016.

[30] Vahedi B., Kuhn W., Ballatore A. (2016). Question-based spatial computing—A case study. Geospatial Data in a Changing World.

[31] Dong E., Du H., Gardner L. (2020). An interactive web-based dashboard to track COVID-19 in real time. Lancet Infect. Dis..

[32] Moran P.A. (1950). Notes on continuous stochastic phenomena. Biometrika.

[33] Mollalo A., Alimohammadi A., Khoshabi M. (2014). Spatial and spatio-temporal analysis of human brucellosis in Iran. Trans. R. Soc. Trop. Med. Hyg..

[34] Mollalo A., Alimohammadi A., Shirzadi M.R., Malek M.R. (2015). Geographic information system-based analysis of the spatial and spatio-temporal distribution of zoonotic cutaneous leishmaniasis in Golestan Province, north-east of Iran. Zoonoses Public Health.

[35] Mollalo A., Blackburn J.K., Morris L.R., Glass G.E. (2017). A 24-year exploratory spatial data analysis of Lyme disease incidence rate in Connecticut, USA. Geospat. Health.

[36] Getis A., Ord J.K. (1992). The analysis of spatial association by use of distance statistics. Geogr. Anal..

[37] Mitchell A. (2005). Spatial Measurements & Statistics.

[38] Kohavi R., John G.H. (1997). Wrappers for feature subset selection. Artif. Intell..

[39] Kursa M.B., Rudnicki W.R. (2010). Feature selection with the Boruta package. J. Stat. Softw..

[40] Nilsson R., Peña J.M., Björkegren J., Tegnér J. (2007). Consistent feature selection for pattern recognition in polynomial time. J. Mach. Learn. Res..

[41] LeCun Y., Bengio Y., Hinton G. (2015). Deep learning. Nature.

[42] Bishop C.M. (1995). Neural Networks for Pattern Recognition.

[43] Hassoun M.H. (1995). Fundamentals of Artificial Neural Networks.

[44] Graupe D. (2013). Principles of Artificial Neural Networks.

[45] Rosenblatt F. (1958). The perceptron: A probabilistic model for information storage and organization in the brain. Psychol. Rev..

[46] Guresen E., Kayakutlu G., Daim T.U. (2011). Using artificial neural network models in stock market index prediction. Expert Syst. Appl..

[47] Mou L., Ghamisi P., Zhu X.X. (2017). Deep recurrent neural networks for hyperspectral image classification. IEEE Trans. Geosci. Remote Sens..

[48] Gardner M.W., Dorling S.R. (1998). Artificial neural networks (the multilayer perceptron)—A review of applications in the atmospheric sciences. Atmos. Environ..

[49] Cascella M., Rajnik M., Cuomo A., Dulebohn S.C., Di Napoli R. (2020). Features, evaluation and treatment coronavirus (COVID-19). StatPearls.

[50] Lai A.G., Pasea L., Banerjee A., Denaxas S., Katsoulis M., Chang W.H., Williams B., Pillay D., Noursadeghi M., Linch D. (2020). Estimating excess mortality in people with cancer and multimorbidity in the COVID-19 emergency. medRxiv.

[51] Hanff T.C., Harhay M.O., Brown T.S., Cohen J.B., Mohareb A.M. (2020). Is There an Association Between COVID-19 Mortality and the Renin-Angiotensin System—A Call for Epidemiologic Investigations. Clin. Infect. Dis..

[52] Alimadadi A., Aryal S., Manandhar I., Munroe P.B., Joe B., Cheng X. (2020). Artificial intelligence and machine learning to fight COVID-19. Physiol. Genom..

[53] Kavanagh N.M., Goel R.R., Venkataramani A.S. (2020). Association of County-Level Socioeconomic and Political Characteristics with Engagement in Social Distancing for COVID-19. medRxiv.

[54] Qu G., Li X., Hu L., Jiang G. (2020). An Imperative Need for Research on the Role of Environmental Factors in Transmission of Novel Coronavirus (COVID-19). Environ. Sci. Technol..

