# Supplementary material for: Artificial Neural Network Modeling of Novel Coronavirus (COVID-19) Incidence Rates across the Continental United States
Source: Int J Environ Res Public Health. 2020 Jun 12;17(12):4204. doi: 10.3390/ijerph17124204 (PMC7344609; doi:10.3390/ijerph17124204)
Supplement: Supplementary file 1 [file ijerph-17-04204-s001.pdf]

All variables and selected variables after feature selection:

| | |
|---|---|
| Dependent variable: COVID-19 incidence | Total number of specialists |
| Median Household Income | Total number of nurse practitioners |
| Income Inequality | Total number of physician assistants |
| Uninsured | Total number of hospitals |
| Unemployment rate | Cardiovascular disease |
| Food Insecurity | Cerebrovascular disease |
| Fair or poor health | Hypertensive heart disease |
| Adult Smoking | Cardiomyopathy and myocarditis |
| Road Density | Atrial fibrillation |
| Particulate matter 2.5 (Min, Max, mean) | Peripheral vascular disease |
| Air quality index (AQI) (Min, Max, mean) | Pancreatic |
| Temperature (Min, Max, mean) | Mesothelioma |
| Precipitation | Hodgkin lymphoma |
| Elevation (Min, Max, Mean) | Leukemia |
| Maximum terrain slope | Tracheal, bronchus, and lung |
| Percent of 65 years and over | Drug use disorder |
| Percent of Asian | Alcohol use disorder |
| Percent of Hispanic | COPD |
| The proportion of African American | Asthma |
| Percent of black males | Interstitial lung disease & pulmonary sarcoidosis |
| Percent of black females | Tuberculosis |
| Percent of white males | HIV/AIDS |
| Percent of white females | Hepatitis |
| Net International migration rate | Lower respiratory infection |
| Total number of primary care physicians | Ischemia |
